# Brazilian Oral Pathology and Oral Medicine: current state of the study of rare diseases

**DOI:** 10.1590/1807-3107bor-2024.vol38.0101

**Published:** 2024-10-28

**Authors:** Renato Assis MACHADO, Daniella Reis Barbosa MARTELLI, Alan Roger SANTOS-SILVA, Hercílio MARTELLI-JÚNIOR

**Affiliations:** (a) Universidade Estadual de Campinas – Unicamp, Piracicaba Dental School, Department of Oral Diagnosis, Piracicaba, SP, Brazil.; (b) Universidade de Montes Claros – Unimontes, Dental School, Department of Oral Diagnosis, Montes Claros, MG, Brazil.; (c) Universidde José do Rosário Vellano – Unifemas, Dental School, Center for Rehabilitation of Craniofacial Anomalies, Alfenas, MG, Brazil.

**To the Editor:**

Rare diseases (RD) have different quantitative and qualitative definitions and variations in classifications. The European Medicines Agency specifies a prevalence of less than 5 in 10,000 subjects (equivalent to less than 1 in 2,000), while in Japan the Ministry of Health, Labour and Welfare defines them as any condition affecting less than 50,000 individuals in the country (equivalent to less than 1 in 2,500 people).^1,2^ In the United States, the prevalence of RD corresponds to 1 in 1,630 subjects considering a current population of about 326 million, although by the time of the Orphan Drug Act of 1983, when the US population was 235.8 million, the prevalence corresponded to 1 in 1,179 subjects. Brazil adopts the definition of the World Health Organization, which establishes a prevalence of 65 cases per 100,000 subjects (1:1,538) (http://portalms.saude.gov.br/saude-de-a-z/doencas-raras). Thus, the disease that is considered rare in one country or continent, may not be rare in another.^2^ It is estimated 13 to 15 million subjects with a RD in Brazil and the participation of Dentistry has been highlighted.^3,4^

Brazil has a population of approximately 215 million people and an extensive territorial area of 8,515,767,049 km^2^. The country is formed by 26 States and a Federal District, which is its capital (Brasília). Moreover, a total of 5,570 municipalities are distributed in five regions (https://www.ibge.gov.br/). The country has 22 Dental Specialties recognized by the Federal Council of Dentistry. Oral Pathology (OP) was recognized as a specialty in 1971 and there are currently 427 registered specialists. Oral Medicine (OM) (Stomatology) was recognized as a specialty in 1992 and has 1,037 registered members (https://website.cfo.org.br/estatisticas/quantidade-geral-de-cirurgioes-dentistas-especialistas/).

In Brazil, there are 98 postgraduate programs (MSc and PhD degrees) in the various branches of Dentistry, and a single institution offers a specific course in OP and OM at Piracicaba Dental School, University of Campinas (FOP-UNICAMP). Twenty-five other institutions have these specialities as concentration areas or research lines in their postgraduate programs in Dentistry (https://sucupira.capes.gov.br/sucupira/public/consultas/coleta/programa/quantitativos/quantitativoAreaConhecimento.jsf;jsessionid=FV9aefthLocXUUKZjTFCWJ2-.sucupira-213?areaAvaliacao=18).

A study conducted in 2016 highlights the main areas of OM in several countries.^5^ The outcomes of the first 50 years of Brazilian OM have been recently described pointing out that among the main aims of the specialty stand out for diagnosing and providing (mostly nonsurgical) treatment of primary diseases of the oral mucosa and the jawbones, as well as salivary gland disorders, orofacial pain, and maxillofacial manifestations of systemic diseases and their medical treatment. Some of these conditions include cancer and oral complications of cancer therapy, infectious diseases, and autoimmune disorders, among others. Also provide comprehensive dental care for patients within a range of complex medical scenarios that impact oral health, including radiation therapy, chemotherapy, bone marrow and solid organ transplantation, molecular targeted therapy in oncology, bone modifying agents and antiresorptive drugs, cardiovascular diseases, acquired immunodeficiency syndrome, and COVID-19.^6^ In general, RD in the field of OM are still little mentioned. Thus, this article aimed to assess the current state of the science concerning RD by Brazilian OP and OM research groups.

Of the 26 postgraduate programs evaluated with the presence of the OP and OM, between March and June 2022, the prevalence of the themes (https://sucupira.capes.gov.br/sucupira/public/consultas/coleta/programa/quantitativos/quantitativoIes.jsf?areaAvaliacao=18&areaConhecimento=40200000) listed in Table 1 was observed. Fifty-one topics were mentioned in the 26 postgraduate programs evaluated. Higher prevalence was observed in the following conditions: Pathogenesis, epidemiology, diagnosis and treatment of diseases of the stomatognathic system; Applied biomaterials; Bioengineering of the stomatognathic system, and oral cancer, representing 34 (66.65%) of the 51 analysed. Only two (3.92%) citations involving malformations and syndromes with orofacial involvement and systemic conditions have been identified.

Each RD, taken separately, affects a limited number of people. Considering, however, that there are up to 8,000 types of RD worldwide when grouped under a single category, their epidemiological impact may become quite significant. Approximately 80% of RD have a genetic etiology. In general, all RD need a multidisciplinary and interdisciplinary team and involvement of the family nucleus of the patient.

To support the importance of the OM in the context of RD, the HPO (Human Phenotype Ontology) system brings over 1,000 genetic syndromes with dental alterations in their clinical spectrum. Among them are: 1) Abnormal dentition (HPO #000164) – 1,049 syndromes; 2) Abnormalities in the shape of the dental crown (HPO #0011091) - 114 syndromes; 3) Taurodontia (HPO #0000679) - 22 syndromes; 4) Macrodontia (HPO #0001572) - 30 syndromes, and 5) Microdontia (HPO #000691) - 167 syndromes.^7^

Important studies in different countries and scientific societies have been carried out analyzing the training activities and performance of OM.^5,6,8-10^ The diagnosis of RD has increased due to multiple factors: (a) the number of RD has increased over time; (b) the population of a given country tends to increase over time; (c) diagnosis continues to improve with the advent of new molecular techniques, and the decreasing cost of next-generation sequencing; and (d) better medical care for several RD should lead to an increase in life expectancy.^2^

In summary, the data presented in this study can lead to reflections on the teaching of RD in undergraduate and graduate courses in Dentistry, as well as the greater participation of scientific societies of OP and OM in this discussion, developing lines of research, presentation at conferences and papers in the area. Furthermore, the participation of both specialties would increase the quality of the phenotypic description of RD, particularly genetic diseases, as well as inform future continuing education courses for dentists.
